# Optimising Connectivity in Deep‐Sea MPA Networks for *Nephrops norvegicus* Recovery in NW Mediterranean Sea

**DOI:** 10.1002/ece3.74447

**Published:** 2026-10-07

**Authors:** Morane Clavel‐Henry, Nixon Bahamon, Miguel López, Jacopo Aguzzi, Joan Navarro, Joan B. Company

**Affiliations:** ^1^ ICM‐CSIC Barcelona Spain; ^2^ GEOMAR Kiel Germany; ^3^ ICATMAR Barcelona Spain

**Keywords:** connectivity metrics, EPT, Fishery No‐take reserves, larval dispersal, marine spatial planning

## Abstract

Marine Protected Areas are essential tools for enhancing population persistence and supporting fisheries through larval dispersal. In the NW Mediterranean, where the Norway lobster *Nephrops norvegicus* (Linnaeus, 1758) is both ecologically and economically important, we assessed the larval connectivity of the existing network of Fishery No‐Take Zones using a 12‐year Lagrangian simulation informed by species‐specific habitat models. Results showed that the current MPA network protects only a small fraction of larval settlement, with connectivity being weak and highly variable across years. Two out of 10 MPAs overlapping with the species habitat emerged as key larval recipients and potential sources, suggesting localised benefits but limited network coherence. To explore improvement strategies, we applied Eigen Perturbation Theory to identify new MPA network configurations based on dispersal dynamics. Both a redesigned and an expanded network significantly enhanced larval settlement and connection persistence, while improving potential recruitment by 8.1% and 4.5% to fishing grounds, respectively. Furthermore, warming trends in the region shortened pelagic larval duration, underscoring the need to integrate climate resilience into MPA design. Our findings highlight the utility of connectivity analyses to guide adaptive spatial planning and highlight the potential to optimise MPA networks for conservation, fisheries management, and climate adaptation.

## Graphical abstract

In the NW Mediterranean, where the Norway lobster ( *Nephrops norvegicus* ) is both ecologically and economically important, larval connectivity in an existing network of Fishery No‐Take Zones was estimated using Lagrangian transport simulation informed by species‐specific habitat models. Two MPAs emerged as key larval recipients and potential sources, suggesting localised benefits but limited network coherence. Both a redesigned and an expanded network significantly enhanced larval settlement and connection persistence, while improving potential recruitment to fishing grounds.

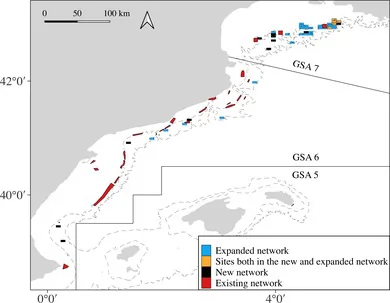

## 1 Introduction

Developing networks of Marine Protected Areas (MPAs) for marine ecosystem conservation and restoration is of utmost importance to reach 30% of the seascape being fully protected by 2030 (Dinerstein et al. 2019; Gill et al. 2024), as called by the EU Biodiversity Strategy (30 × 30; Regulation (EU) 2024/1991) and the United Nations Decade on Ecosystem Restoration. Connectivity has been stated in guidelines for designing MPAs (Burns et al. 2023; IUCN‐WCPA 2008), however the study of connectivity for restoration of fishery and eco‐service is often carried out after implementation, rather than considered while designing the areas. To date, 3.5% of ocean area (http://mpatlas.org/, in August 2026) are covered with strict restriction levels described by the International Union for Conservation of Nature (IUCN), with possibilities of downgrading decisions or having many infractions on them (Roberts et al. 2018; https://atlas.medseaalliance.org). When establishing MPAs, decisions on locations, size, and targeted species are typically guided by multiple objectives, including historical biodiversity data, habitat quality, and socio‐economic aspects (Gaines et al. 2010). Some MPAs even consider species reproductive cycles and implement seasonal closures (van Overzee and Rijnsdorp 2014). However, unlike corridor‐focused designs for migratory species (Kersting and Gallon 2019), MPAs targeting habitat conservation rarely incorporate large‐scale ecological processes that are highly variable or uncertain over time (Gilmour et al. 2022; Meehan et al. 2020). Underlying these dynamics is a transport of species, either through active migration in mobile species or passive dispersal of propagules (e.g., eggs, larvae) in sessile or low‐motile species. For the latter, populations dynamics such as growth, stability, or resilience depend on persistent and successful connections among suitable habitats (White et al. 2019). While better studied in fragmented systems like hydrothermal vents (e.g., Vrijenhoek 2010) or seamounts (e.g., Shank 2010), connectivity patterns in continental margin habitats such as deep‐sea continental slopes remain poorly known despite being among the most impacted by bottom trawl fisheries (Clark et al. 2016; Hiddink et al. 2017).

Spatial connectivity within MPA network can be estimated via modelling of larval dispersal, which benefits from advances in computational capacity, that allow extrapolation of small spatiotemporal variations in a heterogeneous three‐dimensional environment. These models complement molecular tools, which reflect long‐term genetic exchange rather than contemporary dispersal (Jahnke and Jonsson 2022). Studies on larval spatial dispersal are crucial, as MPAs with high recruitment rates have been observed to support greater species densities compared to those with lower recruitment (Wen et al. 2013). Because of their relatively short pelagic life and small control in horizontal displacement, propagules of for example, eggs and larvae, are advected in the ocean by hydrodynamic processes over variable kilometre scales (Pineda et al. 2007). The locations, timing, and success of release (i.e., spawning) and settlement (i.e., when turning to sessile or low‐motile stages) are highly variables due to oceanographic and meteorological conditions, but their trajectories bring insights on the effectiveness of the MPA ecological design (Carr et al. 2017; Lu et al. 2023). More specifically, larval transport studies provide many indicators assessing the standing of a MPA regarding connectivity (dispersal or retention rates; Lu et al. 2023; Jonsson et al. 2020), network theory indicators (Assis et al. 2021; Roberts et al. 2021), spatial mixing (Clavel‐Henry et al. 2024) or providing information on relevant locations (i.e., sources and sinks) for protection measures accounting for connectivity (Melaku Canu et al. 2020).

Spatiotemporal fluctuations in larval dispersal significantly influence population stability (Bani et al. 2018). In marine ecosystems, characterised by high variability, larval connectivity can reflect a complex interplay of oceanographic drivers across multiple time scales, from relatively short‐term disturbances (e.g., storms; Monzón‐Argüello et al. 2012; fronts; Ojeda et al. 2022), to seasonal to interannual events (e.g., atmospheric episodes; Fox et al. 2016), and long‐term trends (e.g., climate change, Coleman et al. 2017). While diverse studies integrate connectivity across multi‐year periods to generate average patterns (e.g., Assis et al. 2021), interannual variability is gaining attention to improve our understanding of underlying processes and assess the robustness of connectivity. Year‐to‐year fluctuations in connectivity strength and occurrence can challenge management goals, though spatial configuration may buffer variability (Harrison et al. 2020). Understanding whether links are persistent (i.e., as consistent exchange of individuals) or episodic informs the design of adaptive management strategies. Persistent links support long‐term conservation planning, while dynamic links may reveal areas sensitive to environmental change. This perspective is especially relevant for deep‐sea and benthic species with slow recovery rates (Clark et al. 2016; Guy et al. 2025). The timescale for characterising temporal variability depends on regional oceanography and the connectivity metrics used (Vogt‐Vincent et al. 2023). While full benefits from MPAs may emerge after a decade (Edgar et al. 2014), certain positive effects, such as biomass spillover and changes in size structure, may appear earlier (Vigo et al. 2023; Quevedo et al. submitted).

The Norway lobster ( *Nephrops norvegicus* ) is a commercially important species (Ungfors et al. 2013), widely exploited across the NE Atlantic, Baltic, and the Mediterranean seas. It is considered an umbrella species for benthic ecosystems (Roberge and Angelstam 2004), as its bioturbation supports sediment oxygenation and remineralization, promoting infaunal diversity and habitat quality (Aguzzi et al. 2023). In the NW Mediterranean, the species is primarily distributed at depths between 300 and 700 m (Vigo et al. 2024). Its stocks presently show signs of overexploitation (Mannini et al. 2023), consistent with trends observed for other key demersal species in the region. To promote stock recovery, France established three demersal Fishery No‐Take Zones (FNTZs) in 2018 (JORF 2018), while Spain designated a network of 27 FNTZs in 2022 (BOE 2022). Among these 30 MPAs, 10 overlap the depth distribution of the Norway lobster, including four that were specifically implemented to support the recovery of the species. To date, only one MPA has been monitored to evaluate the recovery of Norway lobster populations (Vigo et al. 2023).

Given the limited understanding of connectivity performance in this recently established network, our study assesses whether the existing MPAs network design supports ecological connectivity for *N. norvegicus* . We focus on the links among 10 MPAs, which potentially contribute to *N. norvegicus* recovery, and explore larval transport dynamics using a numerical approach based on perturbation theory and connectivity metrics. Our analysis considers cross‐boundary transport and historical knowledge on larval and adult movement ecology, proposing optimised configurations to improve recruitment success and strengthen the long‐term resilience of the *N. norvegicus* populations. The study has supporting insights for management decisions over considering the interannual variability of the environment.

## 2 Methodology

### 2.1 Modelling Approach

#### 2.1.1 Physical Model

Daily hydrodynamic fields for the interannual NW Mediterranean Sea (i.e., horizontal current velocities, water temperature and salinity) were obtained from the open‐access product called ‘Mediterranean Sea Physical Reanalysis’ (Escudier et al. 2020) available via the Marine Copernicus data portal. The dataset covered the period from 15 December to 4 April, for the years 2010 to 2022. Details on the model parametrisation, data assimilation, and validation are described in Escudier et al. (2021). The model domain spans from 38°N to 43.5°N and from 1°W to 7.2°E, encompassing parts of the Gulf of Lion, the Spanish Peninsula from the French border to South of the Ibiza Channel, and the Balearic Islands (Figure 1). The horizontal resolution is around 4–5 km and the vertical structure comprises 141 unevenly spaced layers. This resolution was deemed sufficient to represent offshore hydrodynamics for Lagrangian particle tracking simulations. The Northern Current is well represented, following the continental slope shape from the Gulf of Lion to Ibiza channel, with potential northward deflection along the Balearic Archipelago. In boreal winter, surface temperatures are typically around 13.8°C, no mixed depth layer forms, and current velocities are higher than in summer (García et al. 1994).

**FIGURE 1:**
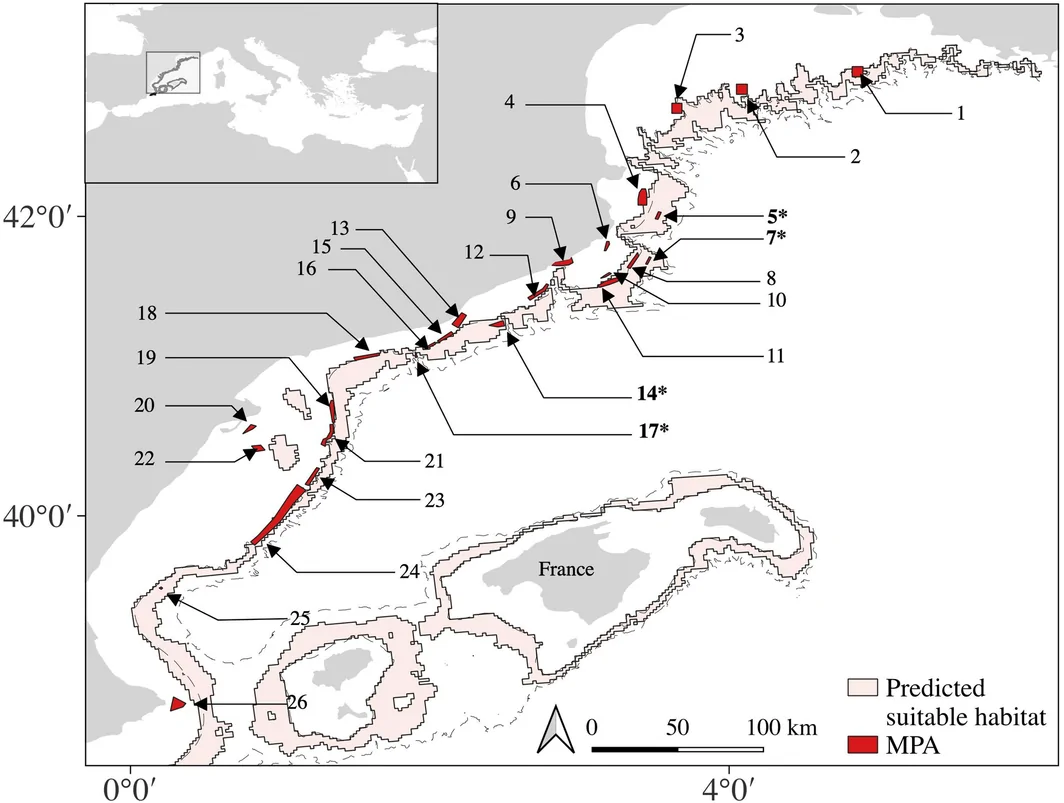
Domain of the study area in the NW Mediterranean Sea, showing the predicted suitable habitat for *Nephrops norvegicus* and the network of permanently closed MPAs. MPAs no. 1, 3, 5, 7, 8, 11, 14, 17, 24, and 25 are located within or adjacent to suitable habitat areas. MPAs no. 5, 7, 14, and 17 specifically target the recovery of *N. norvegicus*. Dashed lines respectively represent the isobaths 200 and 1000 m.

#### 2.1.2 Particle Transport Model

Larval dispersal was simulated using the Lagrangian framework implemented with Opendrift (Dagestad et al. 2018) in Python (version 3.11.11). Each simulation was set up with the release of 566,115 particles, a value that allows to represent 95% of the dispersal variability in our study area (Simons et al. 2013). Particle positions were updated using a 4th order Runge–Kutta scheme, based on hydrodynamic forcings. Horizontal and vertical diffusion were modelled using a random‐walk scheme (Visser 1997), with constant diffusivity coefficients *K*_h_ of 1 m^2^/s and *K*_v_ of 1 *E* ^−5^ m^2^/s, respectively. A time‐step d*t* to of 3 h was used for advection, and 60 s for diffusion. Each particle, representing an individual larvae, was tracked for 45 days, the assumed duration to reach the postlarval at 13°C in the bottom layer. Particle positions were saved daily and collated with environmental variables (e.g., temperature) for post‐simulation analysis. Particles reaching the sea surface or bottom were reflected 50 cm away to avoid boundary effects.

#### 2.1.3 Incorporation of Dynamic Biological Information

In our study, we assumed that the transport duration began after the eggs hatched and stopped after the larvae turned into postlarvae. The individual‐based model (IBM) included life‐history traits of *N. norvegicus* spanning spawning to settling (Table 1), with a particular emphasis on pelagic larval duration (PLD). PLD varied with water temperature and larval stage, following empirical relationships (see Table 1). At each time‐step d*t*, the timer was incremented by d*t*/LD, and when the timer reached 1 (starting from 0), the larvae transitioned to the next stage (Phelps et al. 2015). PLD was computed post‐simulation as the sum of the LD for the three stages and postlarvae duration until settlement in a suitable habitat (see Section 2.3.1). Vertical movement behaviour was stage‐specific and followed patterns reported for *N. norvegicus* larvae (Clavel‐Henry et al. 2024; Smith 1987). Zoea I (Z_I_) ascended to the surface layer and started to perform diel vertical migrations (DVM); Z_II_ remained in the surface layer with DVM; Z_III_ descended towards the bottom. Swimming speeds and directions were stochastically assigned from published distributions (Table 2), and DVM periods were calculated using the ‘astral’ Python package (version 3.2) at 2.17°E (longitude of Barcelona).

**TABLE 1:** Ecological information for parametrisation of the Norway lobster larval transport model.

Early‐life event	Parameter	Observations/Rationales	References	IBM parametrisation
Spawning	Stage development	Spawning females released eggs, which shortly hatched into larvae	Farmer (2009)	Larval stage at *t*(0) is Z_I_
	Day	Mid‐winter in the NW Mediterranean	Sardà (1995); Vigo et al. (2024); Empirical records from ICATMAR (2022a)	15th of December, 1st and 15th of January and February between last trimester of 2010 and first trimester of 2022.
	Time	Around midnight	Moller and Branford (1979)	*t*(0) = 00:00
	Spatial location	Based on fishery logbook and observations		In GSAs 6 and 7, Uniformly random locations in a predicted suitable habitat (López et al. 2026) covering the continental slope of the NW Mediterranean Sea between isobaths of 200 and 700 m (Figure 1).
	Depth	The lobster is a benthic species		Larvae were released at 100 cm from the seafloor.
Drift	Duration	Exponential relationship between Pelagic Larval Duration (PLD), and sea‐water temperature.	Dickey‐Collas et al. (2000)	Z_I_: PLD_I_ = exp.(−0.161*T + 4.265); Z_II_: PLD_II_ = exp.(−0.175*T + 4.646); Z_III_: PLD_III_ = exp.(−0.113*T + 4.188)
	Behaviour	Ontogenetic migration: Z_I_ ascends and then dwells in the surface layers with a diel vertical migration, Z_II_ dwells in a surface layer with a diel vertical migration, Z_III_ descends.	Gage and Tyler (1991); Powell and Eriksson (2013), and herein references	See text below—Random value of swimming velocity from a Gaussian law distribution based on the mean and standard deviation of values given in Table 2—Swimming direction based on a random value from a uniform distribution between 0 and 1, the larval stage, and the larval depth (Table 2)
	Depth of surface layer	Surface depth layer was wide during the day and narrow at night	Lindley et al. (1994); Hillis (1974)	Z_I_ and Z_II_ larval vertical movement were constrained to 0–200 m. (Clavel‐Henry et al. 2024)
Settlement	Stage	Decapodid is benthic, competent for settlement	Powell and Eriksson (2013)	First decapodid

Abbreviation: LD, larval duration.

**TABLE 2:** Swimming direction of the three zoea (Z) and postlarval (PL) stages based on a random value *R* from a uniform distribution between 0 and 1.

Stages	Layer	Below layer	In layer	Above layer
Z_I_	0–200 m	If *R* ≥ 0.2: active ascent; Else: sinking	If *R* ≥ 0.5: active ascent; Else: sinking	—
Z_II_				
Z_III_ and PL	0–1 mab	—	If *R* ≥ 0.5: active ascent; Else: sinking	If *R* ≥ 0.2: active descent; Else: active ascent

Abbreviation: mab. meter above bottom.

Probabilistic swimming directions were established based on larval stage, vertical position, and random values (Table 2). Surface layer depth varied diurnally, following sensitivity tests by Clavel‐Henry et al. (2024). Mortality, feeding, and settlement competency were not modelled due to insufficient data. In total, 60 simulations were performed (five release days × 12 years).

### 2.2 Analysis of Larval Dispersals

#### 2.2.1 Dataset Preparation

Simulation outputs were filtered to retain only individuals that metamorphosed into postlarvae and settled within 1 m of the seafloor inside predicted suitable habitat (Figure 1). Suitable habitat was provided by a joint‐species distribution model (López et al. 2026) calibrated using occurrence records of benthic demersal species (including *N. norvegicus* ) and environmental variables. Model predictions were converted to a binary suitable/unsuitable habitat map for the focus species using a threshold of 0.5 over the continental margin and 0.35 over two distinct patches on the continental shelf (close to MPAs no. 20 and 22 in Figure 1). For each retained trajectory, the youngest settlement age and corresponding coordinates were extracted. Each particle was assigned two identifiers for both its release and settlement positions: (a) the MPA ID (if located within one), and (b) the ID of a grid cell beneath its location. Ten MPAs (e.g., no. 1, 3, 5, 7, 8, 11, 14, 17, 25, and 25) overlapped with *N. norvegicus* suitable habitat, among them MPAs no. 5, 7, 14, and 17 specifically target the recovery of the species (Figure 1). The gridded domain covered 38° to 43.5°N and −2 to 6.2°E, with grid cells measuring 9.13 km (N–S) by 2.56 km (E–W). The resulting dataset contained information on the position, MPA and grid cell at release and settlement, and age of the settled postlarvae.

#### 2.2.2 Selection of Potential MPAs

Candidate areas for protection were identified using Eigen Perturbation Theory (EPT), a method used to analyse connectivity network structure and robustness and optimise site selection (Jacobi and Jonsson 2011). Through dominant eigenvectors, principal patterns of connectivity (i.e., importance of a site as a source and destination) are described, while perturbation analysis assesses how changes in network configuration influence the overall connectivity (Jacobi et al. 2012; Jonsson et al. 2020). A first step involved generating a square, normalised connectivity matrix from grid cells that were occupied by modelled particles and located above suitable habitat within Geographical Sub‐Areas (GSAs) 6 and 7. This matrix represented the cumulative number of larvae dispersing between source and destination grid cells over the 2011–2022 period. The second step involved selecting candidate protected areas using the ‘protectedAreaSelection’ function from the ConnMartTools R package Version 0.3.5. In brief, this function computes eigenvectors of the connectivity matrix to derive a score that quantifies the contribution of each site to network connectivity. Sites are ranked according to this score and the *M* best sites (*M* = 20), which scores are above *θ* threshold (*θ* = 0.05), are selected. Then, first‐order perturbation theory is used to estimate how protecting these sites with a perturbation parameter *δ (δ = 0.1), would affect network connectivity*. Candidate protected areas are obtained by combining the highest‐ranked sites from the connectivity patterns predicted to provide the greatest improvement in overall network connectivity. *M*, θ, and *δ used the default values of the function, but δ = 0.1 was found suitable* (Jacobi and Jonsson 2011).

This approach is used to define an entirely new network in one hand, and an expanded network in another. The EPT was applied on the unfiltered connectivity matrix for estimating the new network, while the connectivity matrix retaining all grid cells connected either as a source or a destination to one of the nine existing MPAs (Figure 1). This latter strategy decreased the size of the connectivity matrix and targeted the reinforcement of larval connectivity within the network.

In a last step, candidate sites were further filtered out using multiyear connectivity matrices. Candidate grid cells were excluded if they did not function as a source connected to any other selected sites throughout the entire 12‐year simulation period (see Figure S1).

#### 2.2.3 Assessment of the MPA Network Performance

Dispersal outcomes were summarised by calculating for the existing, expanded, and new networks of MPAs: (1) the proportion of larvae from MPAs that settled within suitable habitat, (2) mean age of settlement, (3) mean dispersal distance from release to settlement, (4) annual connectivity heatmap, and (5) the spatial density of settled larvae per year.

Temporal (interannual) connectivity among MPAs in existing, expanded and newly‐designed networks was assessed using indicators developed in Clavel‐Henry et al. (2024). These metrics describe the persistence and variability of connections over time, and allow evaluation of each MPA's role in the network. Indicators were computed using only trajectories connecting two MPAs, and reflect the frequency and intensity of larval exchange over the 12‐year simulation.

## 3 Results

### 3.1 Settlement Overview in the NW Mediterranean Sea

The simulations showed that 24%–33% of larvae settled within predicted suitable habitat over the 2011–2022 period, 49.2%–60.7% settled in deeper areas outside of the habitat, and the remaining larvae arrived in shallower areas. From North to South, concentrations of high larval density were found in the Cap de Creus canyons complex, the Palamós Canyon, from the northern margin of Blanes Canyon mouth to the northern widening of the Ebro Shelf (~41°30′N), and the central part of the Ebro continental slope (~40°00′N; Figure 2). High settlement densities were also estimated around the Balearic Islands. However, only a small proportion (2.2%–10.6%) of larvae originated from sources within GSAs 6 and 7. Conversely, depending on the simulated year, 9.6%–36.4% of larvae released from MPAs reached the suitable habitat around the Balearic Islands, with most originating from MPA no. 24.

**FIGURE 2:**
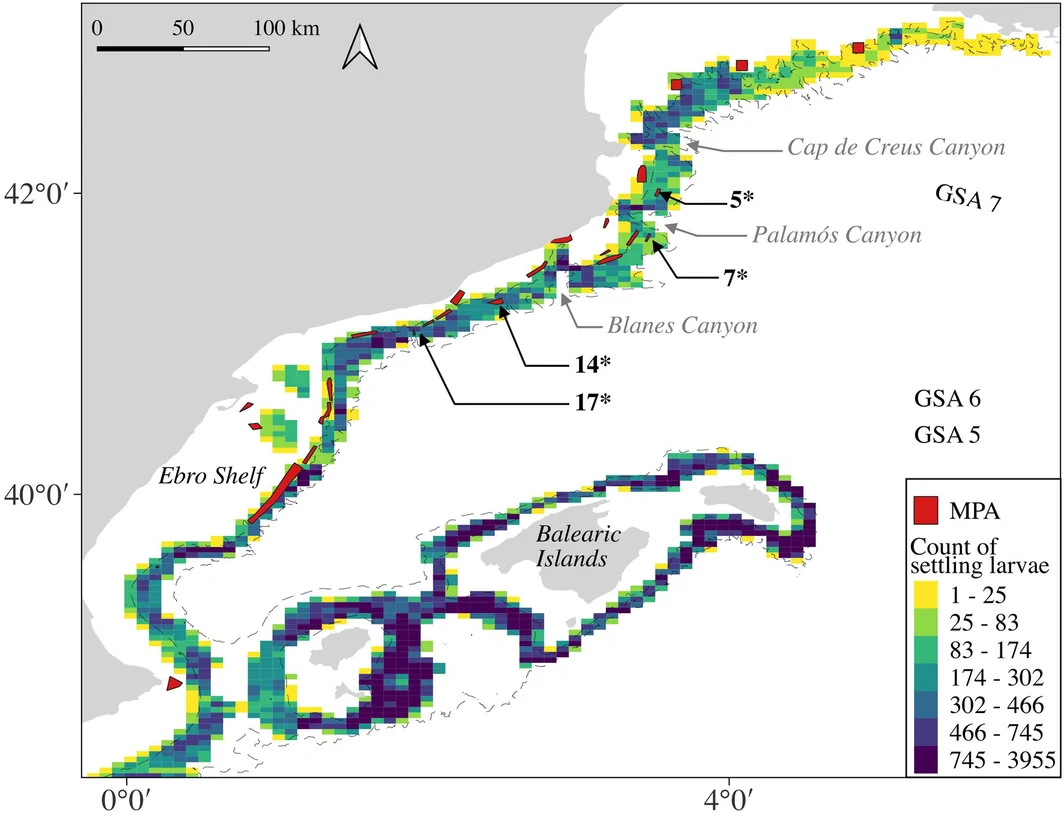
Averaged count of settling postlarvae over the suitable habitat for *Nephrops norvegicus* in the NW Mediterranean Sea from 2011 to 2022. Red polygons indicate permanently closed MPAs. Only relevant MPAs no. 5, 14, and 17 are labelled in the figure (see Section 3.1). Colour scale was discretised based on 6 quantiles.

Regarding settlement within no‐take MPAs, two sites, specifically designated for *Nephrops* protection, stood out for their higher modelled larval density: 20.6 larvae/km^2^ in MPA no. 14 (Barcelona), and 21.3 larvae/km^2^ in MPA no. 17 (Vilanova), surpassing the densities observed in other MPAs. Larval origins were traced from the Gulf of Lion to the widening of the Ebro Shelf, with particularly high source contributions from the continental slope between Cap de Creus and Blanes canyons. This included MPAs no. 5 and 7 (Roses and Palamós), two sites targeting *Nephrops* conservation (see Figures S2 and S3). The location of larval source areas varied greatly across years, even for the two MPAs no. 14 and 17. Sources were identified at a range of latitudinal positions over the study period, reflecting interannual changes in connectivity patterns. Other MPAs exhibited larval density ranging from 0.29 to 6.50 larvae/km^2^. Overall, the existing MPA network accounted for the protection of just 2.8% of the total number of settling larvae.

### 3.2 Location of New Potential MPAs

The selection of potential MPAs using the EPT approach resulted in 30 candidate sites for the expanded network, and 16—two relatively overlapping with MPAs no. 3 and 14—for the new network (Figure 3). Five selected sites were common between the new and expanded network, all located in the GSA 7. The candidate sites were mostly concentrated in the GSA 7 suitable habitat for the expanded network, while they were scattered in GSAs 6 and 7 for candidate sites of the new network. Two candidate sites of the new network were relatively isolated, both located between MPAs no. 25 and 26.

**FIGURE 3:**
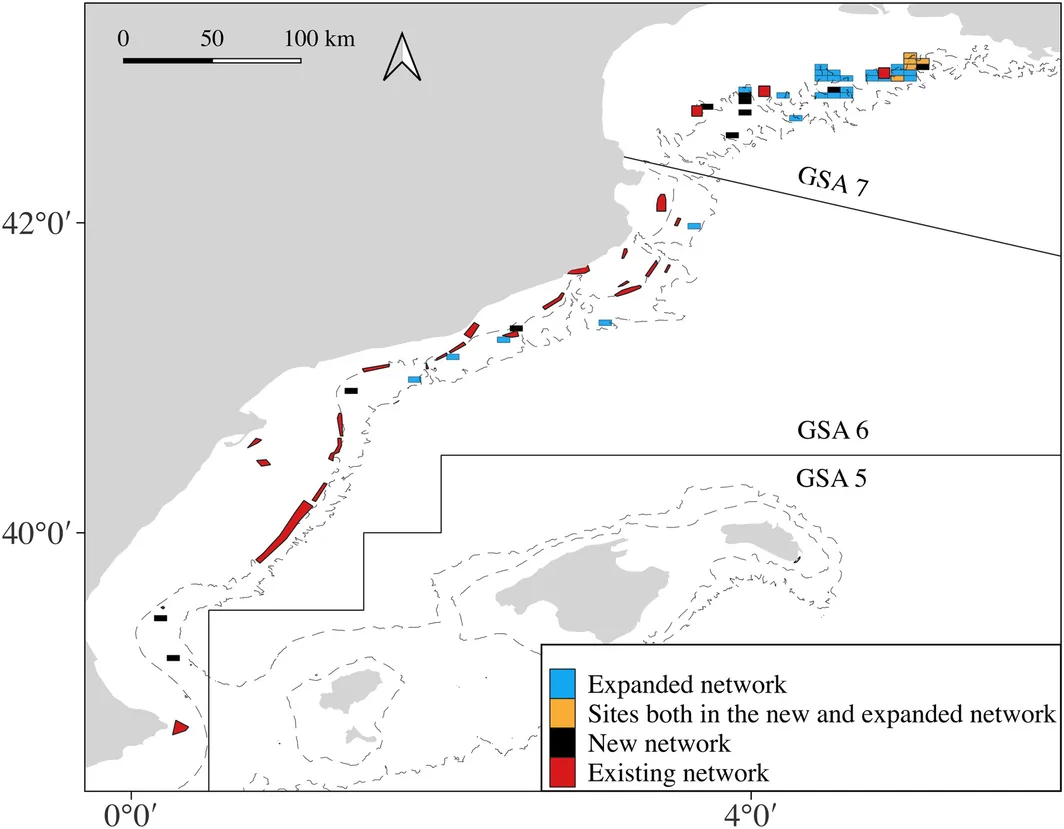
Network of permanent MPAs in the NW Mediterranean Sea (red polygons) and location of suggested MPAs to update the existing networks (blue and orange cells) or to define a new network (black and orange cells) for improving *N. norvegicus* connectivity over time. Purple cells indicate the common suggested MPAs among the existing and new networks.

### 3.3 Overview of the Dispersal From MPAs

From the current MPA network, on average, 36.1% of the larvae reached the predicted suitable habitat. This percentage increased to 40.6% in the expanded network and 44.2% in the new network (Figure 4). Overall, the expanded and new MPA network accounted for the protection of 3.1% and 0.8% of all settling larvae, respectively. Over time, a decrease of approximately 2 days in PLD was observed between 2011 and 2022 for all networks, likely reflecting regional ocean warming (see Table 1). The water temperature during the pelagic larval phase rose by nearly 0.6°C over the same period (Figure S4). Despite the decrease in larval duration, the transport distance per day remained relatively stable, ranging from 3.3 to 8.2 km/day over the 12‐year period.

**FIGURE 4:**
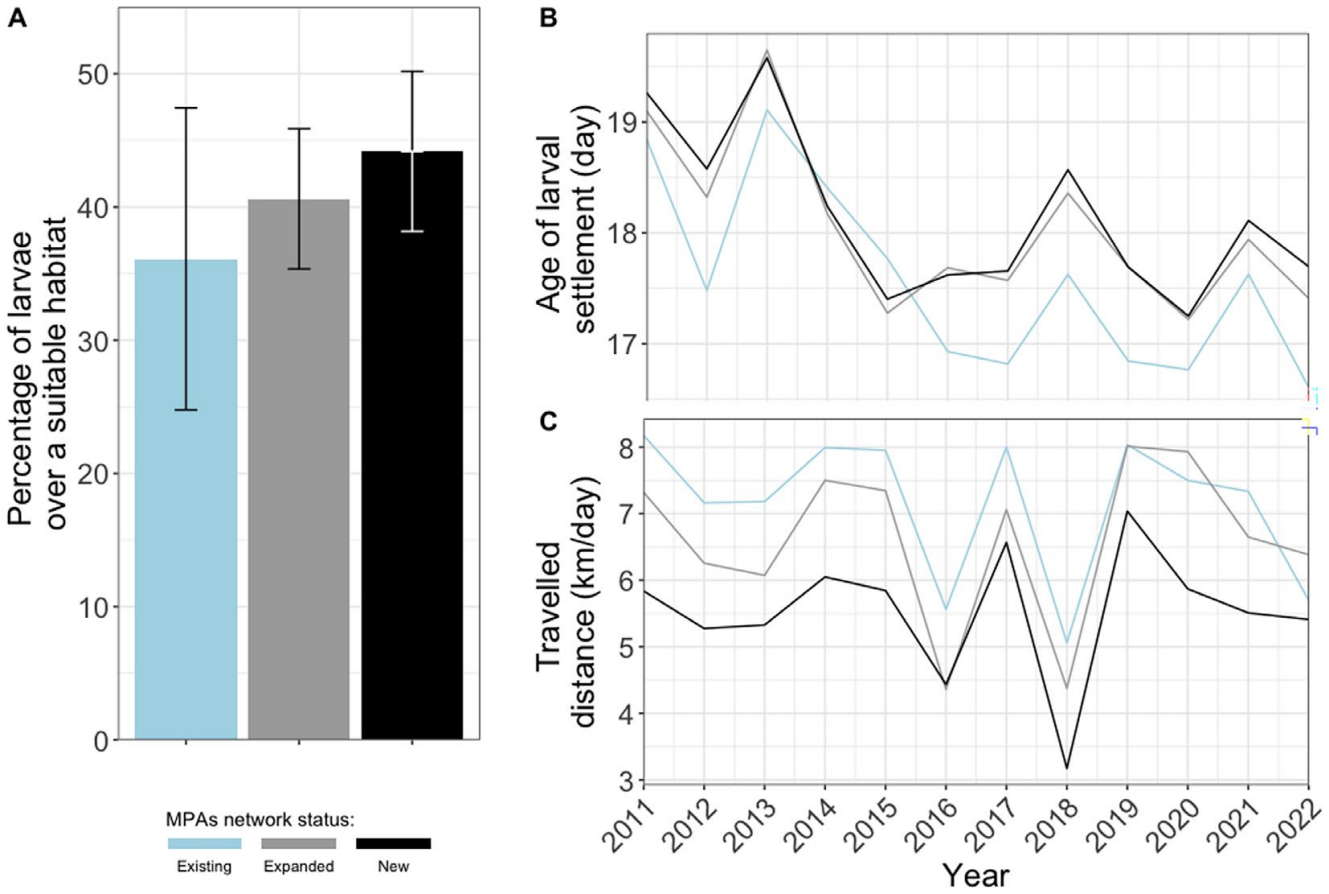
Information about the larval dispersal in relation to the network of MPA sources. (A) General settlement of larvae over a suitable habitat. (B) Age of the larvae at settlement. (C) Distance travelled per day between the spawning and settlement events.

There is a clear spatial impact on density amplitude of settling larvae among the three MPA networks (Figure 5), showing an improvement of settling larvae density with the expanded and new network—notably in the northern part of the study area (> 25 settling larvae per grid cell, Figure 5). In all three networks, larvae settled on the northwestern side of the Balearic Islands and around Ibiza and Formentera, whereas no settlement was predicted along the southeastern side of the archipelago.

**FIGURE 5:**
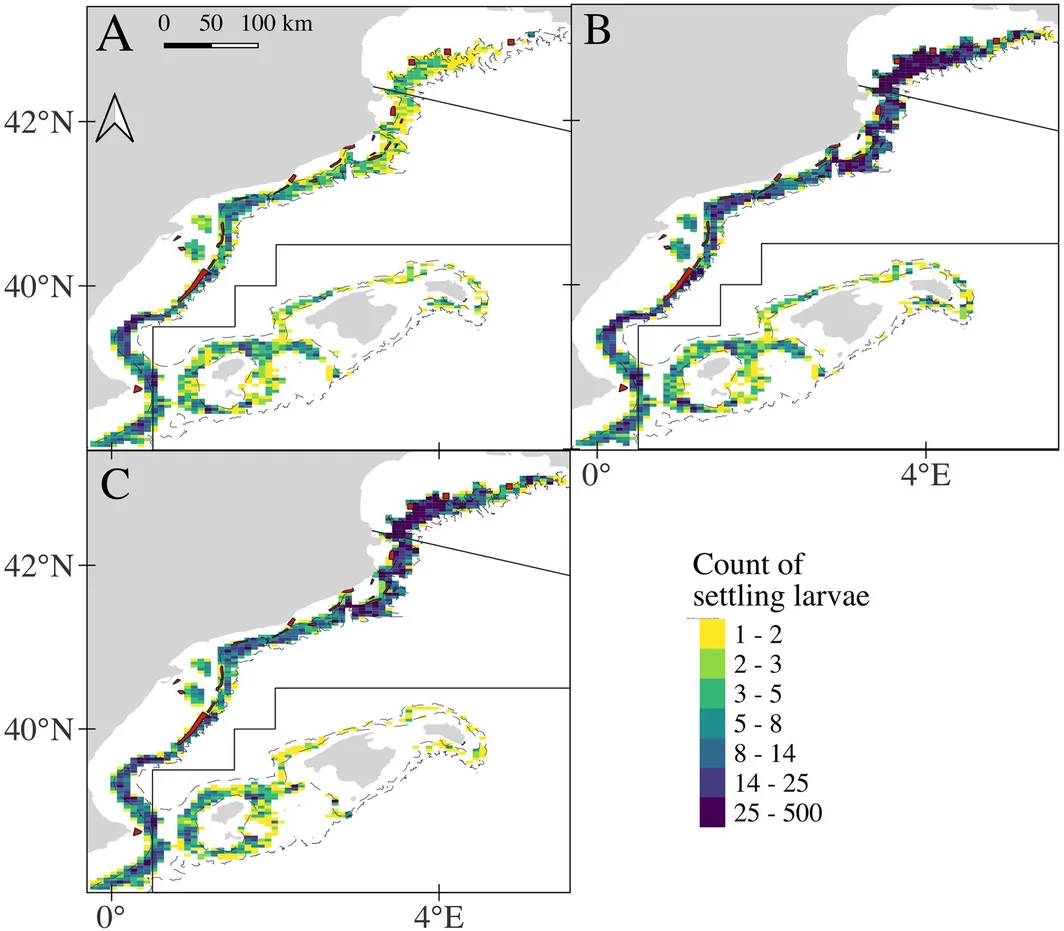
Average count of settled larvae released from MPAs in the existing network (A), in the expanded network (B), and in the new network (C). Colour scale was discretised based on six quantiles.

### 3.4 Temporal Indicators of Connectivity

Comparison of MPA revealed marked differences among individual MPAs and provided a baseline assessment of the existing network. Overall, connectivity was weak, with MPAs maintaining only 1–2.25 connections per year and an average link frequency below 0.3. MPAs no. 14, 17, and 24 stood out for their relatively higher temporal connectivity features. These MPAs maintained links with other MPAs over multiple years, as reflected by a high proportion of repeated links (values > 0.8) and relatively high frequencies of repeated connections (approximately 0.3) (Figure 6A,G). In addition, the particle flow rate of 1 for MPA no. 17 (Figure 6D) indicated little interannual variation in the number of particles transiting through its connection, suggesting relatively stable connectivity over time. In contrast, MPA no. 1, located at the northern edge of the study area, showed no repeated connections. Instead, it was connected to a different MPA in each year (MPA no. 7 in 2012, MPA no. 3 in 2013, MPA no. 14 in 2014, and MPA no. 11 in 2020) resulting in no repeated links across years. This intermittent connectivity, together with similarly low indicator values for MPA no. 21, suggests that both MPAs occupy a peripheral position within the network.

**FIGURE 6:**
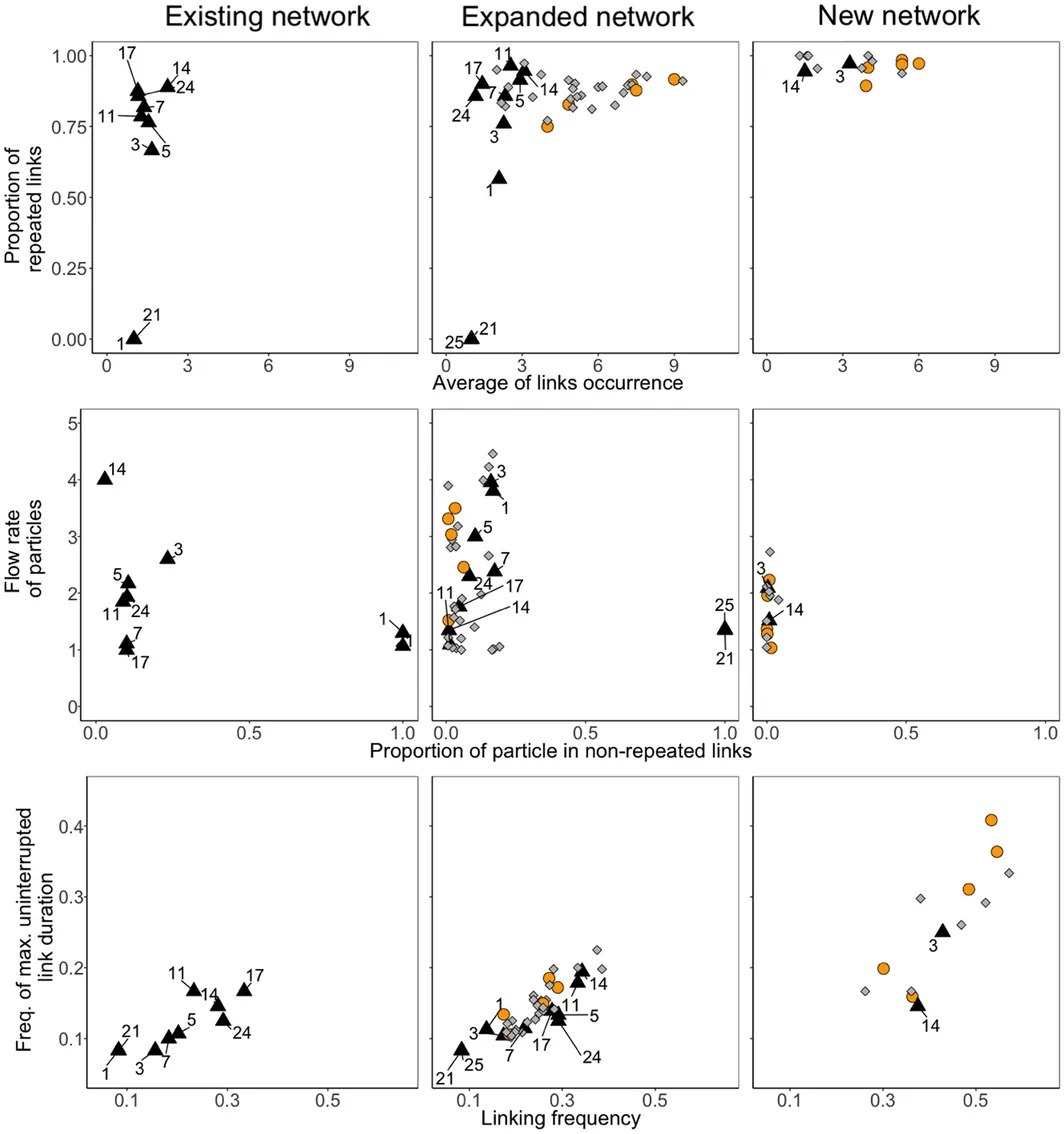
Indicators of temporal connectivity. Symbol shapes and colours denote MPA status: Black triangles represent existing MPAs; orange circles indicate potential protected sites identified by the EPT analysis and included in both the expanded and new networks; grey diamonds represent other potential protected sites.

Both the expanded and new network improved the overall connectivity indicators relative to the existing network, although in different ways. The expanded network substantially increased the number of link occurrence (ranging from 3 to 9 links per year), and increase the probability of repeated link (> 0.5). It also enhanced link frequency for several newly added and existing MPAs, notably MPAs no. 11 and 14 (e.g., > 0.3). In addition, the expanded network slightly improved the connectivity indicators of MPA no. 1, which lies adjacent to a cluster of newly selected sites (Figure 5), by creating new connections between them. In contrast, the new network produce cohesive group of candidate sites with consistently higher connectivity indicator values than those observed in the existing network. The number of annual links ranged from 1.3 to 6, but these links were generally more frequent and more consistently maintained across years. The two existing MPAs retained in the new network (MPAs no. 3 and 14) also exhibited higher probabilities of repeated links than in either the existing or expanded networks. Finally, although the expanded and new networks shared five selected sites, their connectivity characteristics differed. Within the new network, these sites formed fewer but more persistent connections, exhibited lower temporal variability in particle flow (i.e., lower particle flow rate), and showed higher connection frequencies than in the expanded network.

## 4 Discussion

Larval dispersal modelling and studies had importance for assessing the connectivity of the current network of MPAs in the NW Mediterranean Sea together with providing science‐based support for conservation improvement to policy‐makers.

### 4.1 Assessing of *N. norvegicus* Connectivity Within the Current Network

MPA connectivity can be evaluated through three key perspectives: (1) larval settlement within MPAs, (2) larvae export from MPAs, and (3) inter‐MPA larval exchange (Krueck et al. 2017). All three were addressed in this study. First, we observed that only two MPAs located within the predicted suitable habitat received high larval settlement. While modelled densities are relative to our particle release design and not directly comparable to observed *N. norvegicus* abundances (e.g., > 3000 individuals/km^2^ in MPA no. 5; Vigo et al. 2023), these results suggest that connectivity may contribute to the success of these MPAs. In particular, MPA no. 5 appear to be both a settlement site and a larval source, supporting the hypothesis that key MPAs may drive broader population resilience of the MPAs, as suggested by Ospina‐Alvarez et al. (2020). Confirming this would require molecular analyses and targeted field sampling, which remain logistically challenging in no‐take areas.

Second, our results show that MPAs contribute to larval recruitment within fished areas. An estimated 36.1% of larvae released from MPAs settled in suitable grounds including areas of high fishing effort (e.g., northern Blanes Canyon, Vigo et al. 2024; ICATMAR 2022b). While overall larval input from MPAs remains modest relatively to non‐protected sources, these findings suggest that MPAs may act as refuges and potential population reservoir, especially under scenarios of local stock collapse. On another hand, MPAs also appeared to supply relatively large amount of larvae to grounds of the Balearic Islands. Reciprocal connectivity, i.e., from Balearic to the Spanish peninsula was not analysed in our simulations, although evidenced in others (Wesselmann et al. 2018). This pattern likely reflects circulation splitting patterns, such as at the North Balearic Front, and Eivissa Channel (Clavel‐Henry et al. 2021), previously linked to gene flow in other species (Wesselmann et al. 2018; Andrello et al. 2013).

Third, although the network showed an overall southward connectivity trend, interannual variability was high. Most MPAs maintained few and inconsistent connections, with no site establishing stable links throughout the 12‐year period. MPAs no. 14, 17, and 24 stood out for higher temporal persistence. In the case of MPA no. 24, its larger surface area (~225 km^2^) may have increased the probability of establishing repeated connections over time. For MPAs no. 14 and 17, however, their favourable indicators are more plausibly linked to their oceanographic settings and geographical location: they lie in a region influenced by retentive mesoscale features such as eddies (Sabatés et al. 2007). The indicators of temporal connectivity, assessed from a source perspective, underline the importance of location in sustaining functional connectivity.

### 4.2 Connectivity Assessment to Support Management Decisions

#### 4.2.1 Network Improvement

Connectivity analyses offer a valuable framework to guide the design and improvement of MPA networks. In our study, both an expanded and a redesigned network, based on Eigen Perturbation Theory, yielded significant connectivity performance relative to the existing configuration. The redesigned network increased larval settlement in suitable habitat by 8.1%, whereas the expanded network, which added protected sites near existing MPAs, achieved a 4.5% increase. In the scenario with a fully redesigned network, the EPT identified 16 candidate protected sites, representing six additional sites compared with the 10 existing MPAs overlapping the species' habitat. Among these, two sites were identified as key stepping stones linking the northern and southern parts of the network. This new configuration led to increased larval settlement within suitable habitat and enhanced the likelihood of sustained connectivity between protected sites through time. Although a full redesign may be scientifically optimal, implementation is constrained by political and socio‐economic considerations. Many of the new candidate sites, for example, are located in French waters, raising jurisdictional challenges if the conservation benefits primarily support Spanish fisheries.

A realistic alternative may involve merging the redesigned and expanded networks, then filtering for high‐performing sites. This would allow prioritisation of a smaller number of MPAs with the highest conservation impact. Importantly, both network configurations also improved connectivity to fishing grounds, suggesting well‐designed MPA can provide ecological and socio‐economic benefits. Despite connectivity rarely being a primary consideration in NW Mediterranean planning, our finding highlight that even modest adjustments can substantially enhance network performance. More broadly, the proposed EPT framework is readily transferable to other species for which connectivity matrices can be generated (e.g., blue mussel, Berglund et al. 2012; dusky grouper, Andrello et al. 2015). Extending the approach to multiple species would support the design of MPA networks that better represent regional biodiversity and connectivity patterns.

#### 4.2.2 Consideration for Integrating Climate Change

The NW Mediterranean Sea is undergoing rapid environmental change, including warming trends of nearly 0.6°C over the past decade (Bahamon et al. 2020; Margirier et al. 2020). In our simulations, this warming corresponded with a two‐day reduction in the pelagic larval duration of *Nephrops*, although mean dispersal distance remained stable. Given the high interannual variability in connectivity, driven largely by mesoscale circulation, it remains challenging to isolate temperature effects alone. However, ongoing changes in regional hydrodynamics, particularly those in the northern Balearic Sea (Parras‐Berrocal et al. 2024), may alter key dispersal pathways. Some MPAs, especially those in the southern part of the network (e.g., MPA no. 14–25), could become more vulnerable if future circulation changes disrupt existing retention or connection patterns (Clavel‐Henry et al. 2021). Connectivity analyses can help anticipate such scenarios by identifying persistent larval settlement areas, potential climate refugia, that may retain favourable conditions despite environmental change. Climate change is expected to shift species distributions either poleward or to greater depths, as organisms respond to changing thermal and ecological conditions (Poloczanska et al. 2013). This could reduce the effectiveness of existing MPAs, particularly for species with narrow habitat requirements, low adults motility, or limited dispersal capacity (Champion et al. 2024). To address this, climate‐resilient MPA design is advocated (O'Regan et al. 2021), which includes identifying sites likely to remain robust, become vulnerable, or function as future refugia. Connectivity studies, particularly when incorporating projected environmental changes, can support such effects by identifying stable larval settlement. This approach offers valuable foresight for adaptive MPA planning in the context of a warming Mediterranean Sea.

## 5 Conclusion

This study provides the first connectivity‐based assessment of the *Nephrops norvegicus* MPA network in the NW Mediterranean Sea. Results reveal that the current network protects only a small fraction of larval settlement, with MPA connectivity limited in both strength and persistence within the network. Using connectivity‐informed design, we show that strategic redesign or targeted expansion, based on EPT‐based site selection, can substantially increase larval settlement within suitable habitats and improve temporal stability of connections. These insights offer promising avenues to strengthen the resilience of *Nephrops* populations. Our findings support the use of connectivity analyses in MPA planning for Norway lobster and other species in a changing Mediterranean environment and highlight the potential to reconcile conservation objectives with fishery recovery and climate resilience. Even within jurisdictional and practical constraints, targeted improvements to MPA networks can produce measurable ecological benefits and support long‐term population sustainability.

## Author Contributions

**Morane Clavel‐Henry:** conceptualization (lead), data curation (lead), formal analysis (lead), investigation (lead), methodology (lead), visualization (lead), writing – original draft (lead), writing – review and editing (equal). **Nixon Bahamon:** conceptualization (supporting), funding acquisition (lead), project administration (lead), writing – original draft (equal), writing – review and editing (equal). **Miguel López:** conceptualization (supporting), formal analysis (supporting), investigation (supporting), writing – original draft (equal), writing – review and editing (equal). **Jacopo Aguzzi:** funding acquisition (lead), project administration (lead), writing – original draft (equal), writing – review and editing (equal). **Joan Navarro:** funding acquisition (supporting), project administration (supporting), writing – original draft (equal), writing – review and editing (equal). **Joan B. Company:** funding acquisition (lead), project administration (lead), writing – original draft (equal), writing – review and editing (equal).

## Funding

This work was supported by Ministerio de Ciencia, Innovación y Universidades, FPU21/01366, PID2020‐114732RB‐C32, PLEC2021‐007525/AEI/10.13039/501100011033, and Department of Climate Action, Food and Rural Agenda of the Generalitat de Catalunya, BOE‐A‐2023‐25106, and European Maritime and Fisheries Fund EMFF.

## Conflicts of Interest

The authors declare no conflicts of interest.

## Supporting information


**Figure S1:** Conceptual methodology for potential protected sites selection using the Eigen Perturbation Theory approach. The example is limited to 4 years for illustration purposes.
**Figure S2:** Average distribution of larval sources reaching the MPA no. 14 over 2011–2022.
**Figure S3:** Average distribution of larval sources reaching the MPA no. 17 over 2011–2022.
**Figure S4:** Average temperature conditions during the pelagic life of *N. norvegicus* and based on three network situations.

## Data Availability

Data created and processed for this publication can be found in the Zenodo repository. Scripts for the transport model parametrisation (https://github.com/MoraneC/Nephrops_IBM_files) and for getting the outcomes described in the publication (https://github.com/MoraneC/RScripts) are available in Github.
