# Supplementary material for: Optimising Connectivity in Deep‐Sea MPA Networks for Nephrops norvegicus Recovery in NW Mediterranean Sea
Source: Ecol Evol. 2026 Oct 7;16(10):e74447. doi: 10.1002/ece3.74447 (PMC13643509; doi:10.1002/ece3.74447)
Supplement: Supplementary file 1 [file ECE3-16-e74447-s001.docx]

**Supplementary material**

Figure S1. Conceptual methodology for potential protected sites selection using the Eigen Perturbation Theory approach. The example is limited to 4 years for illustration purposes.

Figure S2. Average distribution of larval sources reaching the MPA nº14 over 2011-2022.

Figure S3. Average distribution of larval sources reaching the MPA nº17 over 2011-2022.

Figure S4. Average temperature conditions during the pelagic life of *N. norvegicus* and based on three network situations.
